# Succinylcholine-induced rhabdomyolysis in a patient with *RYR1* and *BCHE* variants: A case report

**DOI:** 10.17305/bb.2025.13435

**Published:** 2025-11-10

**Authors:** Tracy E Harrison, Toby N Weingarten, Juraj Sprung

**Affiliations:** 1 Department of Anesthesiology and Perioperative Medicine, Mayo Clinic, Rochester, MN, USA

**Keywords:** Butyrylcholinesterase deficiency, rhabdomyolysis, ryanodine receptor variant, skeletal muscle

## Abstract

Masseter muscle spasm after succinylcholine can herald malignant hyperthermia (MH) in genetically susceptible individuals. We aimed to describe the perioperative course and genetic findings in a patient who developed transient masseter spasm and postoperative rhabdomyolysis after general anesthesia. This single-patient case report draws on perioperative observations, laboratory testing, and whole-genome sequencing. Immediately after induction with propofol and succinylcholine, the patient experienced transient masseter spasm; anesthesia was then maintained with total intravenous anesthesia (propofol and remifentanil). Postoperatively, laboratory studies showed severe rhabdomyolysis with mild pigment nephropathy; the patient received intravenous hydration, laboratory values normalized by postoperative day 4, and discharge occurred in good condition. Whole-genome sequencing identified heterozygous ryanodine receptor 1 (*RYR1)* c.1840C>T (p.Arg614Cys)—a known MH-susceptibility variant in the skeletal-muscle ryanodine receptor—and butyrylcholinesterase (*BCHE)* c.293A>G (p.Asp98Gly), which reduces butyrylcholinesterase activity and delays succinylcholine hydrolysis. The coexistence of these variants likely synergistically increased sarcoplasmic reticulum Ca^2+^ release and prolonged succinylcholine effect, precipitating rhabdomyolysis; to our knowledge, this appears to be the first reported case linking concurrent *RYR1* and *BCHE* variants to rhabdomyolysis following general anesthesia.

## Introduction

Ryanodine receptor 1 (RYR1) is a large calcium-release channel situated in the sarcoplasmic reticulum membrane of striated skeletal muscle. The calcium channel protein complex, which plays a critical role in muscle excitation–contraction coupling, consists of RYR1 and the dihydropyridine receptor (DHPR), an L-type voltage-gated calcium channel located on the T-tubular sarcolemmal membrane. Following depolarization of the muscle membrane, induced by acetylcholine or succinylcholine, DHPR undergoes a conformational change that activates RYR1, leading to calcium release from the sarcoplasmic reticulum. Genetic variants of the RYR1 protein can result in excessive cytosolic calcium after depolarization, potentially causing oxidative overload, myopathy, and skeletal muscle breakdown.

Variants of *RYR1* are implicated in a diverse range of inherited muscle disorders, including malignant hyperthermia (MH) [1, 2]. MH is a pharmacogenetic, life-threatening hypermetabolic reaction triggered by exposure to halogenated anesthetics and succinylcholine [3, 4]. Halogenated agents interact directly with the RYR1 channel, while succinylcholine exerts its effects indirectly by binding to nicotinic acetylcholine receptors (nAChRs). This interaction induces membrane depolarization, subsequently triggering calcium release from the sarcoplasmic reticulum via RYR1.

In patients with pathogenic *RYR1* variants, calcium efflux into the cytosol following succinylcholine administration can be excessive, resulting in sustained muscle contraction, myofibrillar disruption, and rhabdomyolysis [1]. We present a case of a patient who experienced mild masseter spasm following succinylcholine during anesthetic induction, with subsequent development of severe rhabdomyolysis in the postanesthesia care unit (PACU), suggesting an underlying *RYR1*-related disorder. Whole-genome sequencing later identified two variants that likely acted synergistically to produce this severe clinical presentation.

## Case report

The Mayo Clinic Institutional Review Board does not require review of single-patient cases. A 48-year-old otherwise healthy man with hearing loss secondary to medial canal fibrosis underwent hearing aid insertion under general anesthesia. Anesthesia was induced with intravenous propofol and succinylcholine. Mild masseter muscle spasm was observed following induction; however, after the administration of additional propofol, mouth opening improved, allowing for successful tracheal intubation. The presence of masseter spasm raised concerns regarding MH susceptibility. Consequently, anesthesia was maintained using total intravenous anesthesia with propofol and remifentanil. Throughout the procedure, core temperature, end-tidal carbon dioxide, and heart rate were closely monitored and remained within normal limits. At the conclusion of the two-hour surgery, the patient’s trachea was extubated, and he was transferred to the PACU. Initial recovery was unremarkable, with discharge criteria met within 60 min.

**Figure 1.**
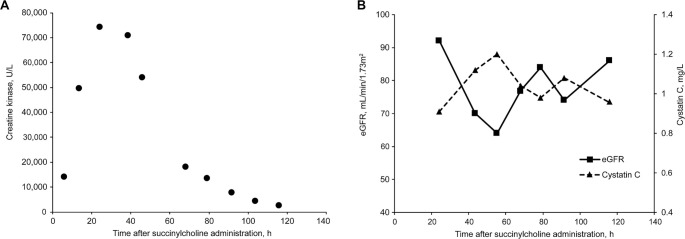
**Biomarkers of rhabdomyolysis following succinylcholine administration in a patient with *BCHE* and *RYR1* variants.** (A) Serum creatine kinase levels; (B) Serum cystatin C levels and cystatin C–estimated glomerular filtration rate (eGFR).

Immediately prior to discharge, the patient reported passing “black-colored urine,” raising concerns for rhabdomyolysis. Laboratory tests performed six hours after succinylcholine administration revealed a serum potassium level of 4.6 mmol/L (reference: 3.6–5.2 mmol/L), serum creatine kinase (CK) of 14,143 U/L (reference: 39–308 U/L), and urine myoglobin >5000 µg/L (reference: ≤65 µg/L). Given the diagnosis of rhabdomyolysis with myoglobinuria, the patient was admitted for intensive intravenous hydration and observation. At 14 h post-succinylcholine administration, the patient’s CK level had risen to 49,884 U/L, peaking at 74,320 U/L at 24 h (Figure 1A). To evaluate potential kidney injury, serum creatinine and cystatin C levels were serially monitored. Serum creatinine remained stable (0.94–1.16 mg/dL), while serum cystatin C, a non-glycosylated protein used to estimate glomerular filtration rate (GFR), increased from 0.91 mg/L on postoperative day 1–1.20 mg/L on day 2 (reference: 0.63–1.03 mg/L) (Figure 1B). This corresponded to a decline in estimated GFR from 92 to 64 mL/min/1.73 m^2^, consistent with mild pigment nephropathy. Serum alanine aminotransferase level was 63 U/L in the PACU and transiently increased to 228 U/L on postoperative day 2 (reference: 7–55 U/L). Serum aspartate aminotransferase level was 144 U/L in the PACU and transiently increased to 818 U/L on postoperative day 2 (reference: 8–48 U/L). The preoperative platelet count was 264 × 10^9^/L, which decreased to 75 × 10^9^/L on postoperative day 1 and rebounded to 275 × 10^9^/L on postoperative day 2 (reference: 135–317 × 10^9^/L). The white blood cell count was 17.2 × 10^9^/L on postoperative day 1 (reference: 3.4–9.6 × 10^9^/L). Hepatitis panels (A, B, and C) were all negative. The patient was discharged on postoperative day 4, with all laboratory values returning to the normal range.

### Genetic counseling and testing

Given the suspicion of MHS, the patient was referred for genetic evaluation. A clinical genomics consultant conducted a comprehensive assessment, gathering additional details of the patient’s medical history that had not been documented prior to the procedure. Through directed questioning, the patient reported experiencing intermittent muscle spasms and pain following physical exertion, particularly in warm environments. These episodes were primarily localized to the abdominal muscles but occasionally involved generalized muscle cramping affecting multiple muscle groups. The patient described the spasms as transient and self-limited, occurring mainly after strenuous activity, without associated weakness or other systemic symptoms. No formal evaluation for these symptoms had been conducted previously. The patient also reported a history of two surgical procedures performed under general anesthesia in his hometown; however, specific information regarding the anesthetic agents used was unavailable. The first procedure, an appendectomy at age 18, was reportedly uncomplicated. Following the second procedure, an ambulatory otologic surgery at age 39, the patient experienced dark urine, myalgia, and abdominal muscle cramps within 24 h post discharge. All symptoms resolved spontaneously within one day, leading the patient not to seek medical evaluation or inform the treating physician at that time. Additionally, the patient noted a family history of similar, albeit milder, symptoms: his father and two older brothers occasionally experience muscle cramping, particularly after physical exertion.

In light of this clinical history, rapid whole-genome sequencing was performed at the Rady Children’s Institute for Genomic Medicine Clinical Laboratory (Clinical Genome Center, San Diego, CA, USA), as this testing is not available at Mayo Clinic. Whole-genome sequencing identified two heterozygous gene variants. The first was a c.1840C>T variant in the *RYR1* gene, resulting in a missense mutation that substitutes arginine with cysteine at position 614 (p.Arg614Cys). This substitution is consistent with *RYR1* receptor-related disorders and is strongly associated with MHS [1, 2]. The second variant was a 293A>G in the gene encoding *BCHE*, resulting in an aspartic acid to glycine substitution at position 98 of *BCHE* (c.293A>G; p.Asp98Gly). This substitution causes *BCHE* deficiency, which slows the hydrolysis of choline esters (e.g., succinylcholine) and leads to prolonged succinylcholine-induced muscle cell membrane depolarization. The specific genetic constellation of these two variants likely contributed to the phenotypic presentation in our patient. Specifically, the pathogenic effects of the *RYR1* variant on skeletal muscle breakdown were likely exacerbated by sustained cell membrane depolarization caused by delayed succinylcholine degradation due to *BCHE* deficiency. Comprehensive genetic counseling was provided, emphasizing preventive measures to reduce the risk of recurrent rhabdomyolysis and advising the patient to wear an MH susceptibility alert bracelet.

### Ethical statement

Written informed consent has been obtained from the patient to publish this report. The principles outlined in the Declaration of Helsinki were followed.

## Discussion

**Figure 2.**
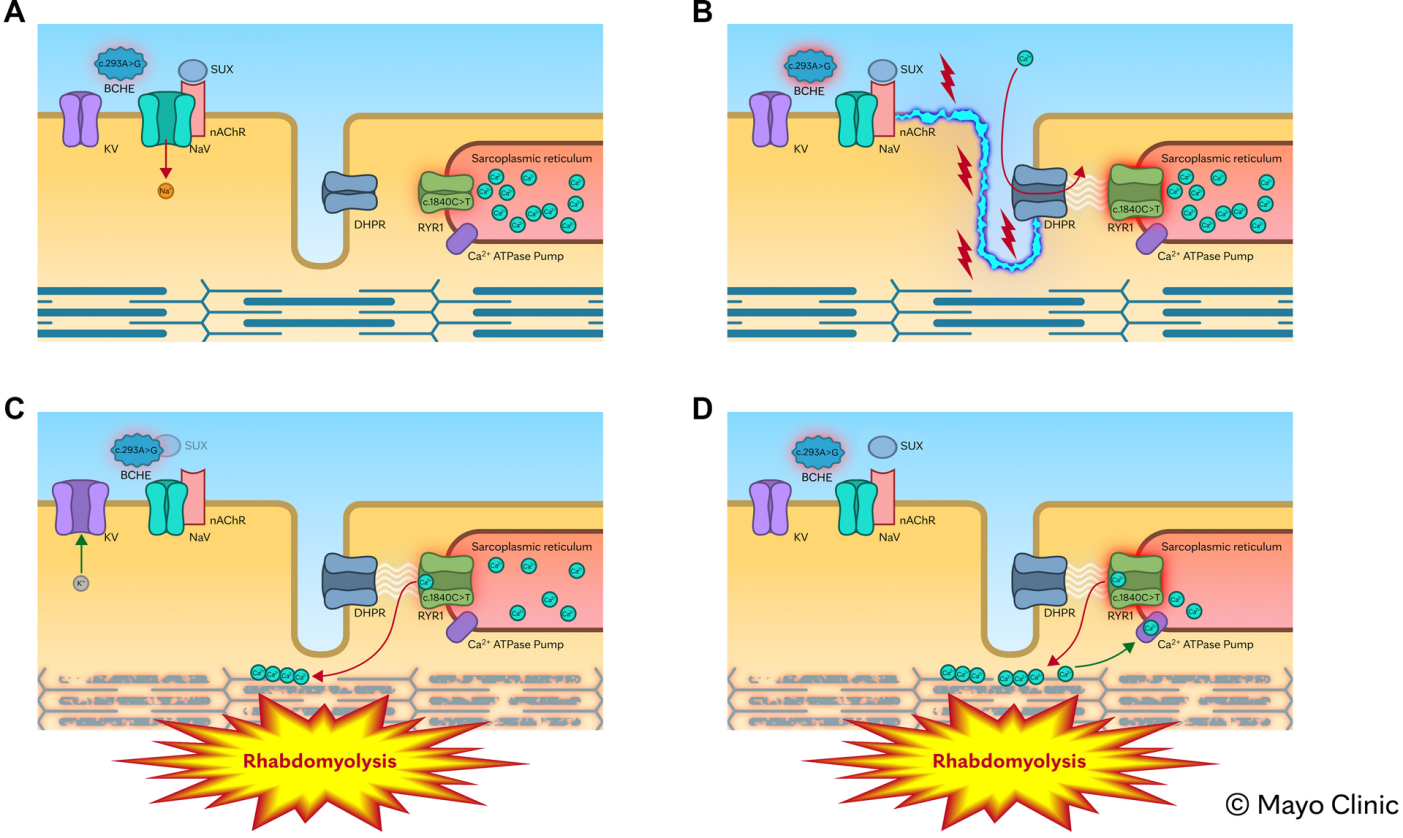
**Proposed mechanism of severe rhabdomyolysis in a patient with *BCHE* deficiency and an *RYR1* pathogenic variant.** (A) Key components involved in skeletal muscle contraction are illustrated. (B) Succinylcholine (SUX) binds to nAChRs, triggering the opening of voltage-gated sodium channels (NaV) and initiating skeletal muscle membrane depolarization (blue serrated line). This depolarization activates the DHPR, which subsequently opens the *RYR1* calcium channel on the sarcoplasmic reticulum membrane. In normal muscle, *RYR1* opens briefly to release calcium into the cytosol, then closes to allow relaxation. However, in the presence of an *RYR1* pathogenic variant [c.1840C>T (p.Arg614Cys)], the channel remains abnormally open, leading to excessive and prolonged calcium release, sustained muscle contraction, and eventual rhabdomyolysis. (C and D) In a patient with butyrylcholinesterase (*BCHE)* deficiency [c.293A>G (p.Asp98Gly)], the metabolism of SUX is impaired, leading to delayed breakdown. This prolongs depolarization of the muscle membrane, which in turn causes sustained activation of the RYR1. This further enhances calcium leakage into the cytosol, perpetuating sustained myofibrillar contraction and contributing to severe muscle damage with rhabdomyolysis. (Used with permission of Mayo Foundation for Medical Education and Research.)

This case report describes the simultaneous presence of pathogenic variants in the *RYR1* and *BCHE* genes in a patient who developed mild masseter spasm after receiving succinylcholine, followed by severe rhabdomyolysis and mild pigment nephropathy. We propose that the coexistence of *BCHE* deficiency and a pathogenic *RYR1* variant synergistically contributed to the severity of rhabdomyolysis. Impaired succinylcholine hydrolysis resulting from *BCHE* deficiency likely prolonged muscle membrane depolarization, permitting sustained calcium release from the sarcoplasmic reticulum into the cytosol. This prolonged calcium efflux may have triggered persistent muscle contraction and extensive myofibrillar breakdown. Figure 2 illustrates the proposed pathophysiologic pathway underlying the rhabdomyolysis observed in this patient, with animations of the mechanism provided in the Supplemental data.

### Biochemical abnormalities in our patient with rhabdomyolysis

Our patient demonstrated several biochemical abnormalities, most notably a transient rise in serum transaminases. The increased transaminase levels were likely due to muscle breakdown [5] rather than damage to the hepatic parenchyma, further supported by negative hepatitis panels. Another abnormal finding was thrombocytopenia. While this can arise from various causes, including hemodilution due to overhydration, the most plausible explanation in this case relates to rhabdomyolysis. During rhabdomyolysis, myoglobin-derived heme released from damaged muscle tissue is converted to hemin in the bloodstream [6]. Hemin has been shown to induce platelet activation and subsequent consumption, leading to a reduction in platelet count [7, 8]. The patient also exhibited leukocytosis, a response associated with inflammatory stress (release of epinephrine, norepinephrine, and/or inflammatory cytokines such as IL-6 and TNF-alpha) related to muscle tissue content release into the bloodstream [9]. However, other etiologies, such as volume depletion with hemoconcentration, may also be contributory.

### Prevalence of *RYR1* and *BCHE* genetic variants in the general population

The prevalence of pathogenic *RYR1* variants in the general population is estimated at approximately 1 in 300 individuals [10]. The *RYR1* c.1840C>T (p.Arg614Cys) variant, specifically associated with MHS, has an allele frequency of about 1 in 5000 [11]. Due to the incomplete penetrance of many *RYR1* variants and the necessity of exposure to triggering agents (e.g., volatile anesthetics or succinylcholine) for clinical manifestations, the reported incidence of MH is significantly lower, ranging from approximately 1 in 10,000 to 1 in 30,000 anesthetic events in children [12], and from 1 in 50,000 to 1 in 100,000 in adults [13, 14]. Heterozygous *BCHE* deficiency occurs in about 1 in 25 to 1 in 50 individuals [15]. Since these genetic variants represent independent pathophysiological processes, the likelihood of both occurring in the same individual can be estimated by multiplying their respective frequencies. Based on published allele frequency data, the estimated prevalence of coexisting *RYR1* c.1840C>T (p.Arg614Cys) and heterozygous *BCHE* c.293A>G (p.Asp98Gly) variants in a single individual ranges from approximately 1 in 125,000 to 1 in 250,000. To our knowledge, this is the first reported case of a patient harboring both variants and presenting with rhabdomyolysis following exposure to a triggering anesthetic agent.

The pathogenic *RYR1* variant identified in our patient has been associated with MH and other *RYR1*-related myopathies [16–19]. While numerous single-nucleotide variants in genes linked to skeletal muscle calcium release have been documented [16, 20, 21], the only variants positively correlated with MHS are located in the *RYR1* (19q13.2), *CACNA1S* (1q32.1), and *STAC3* (12q13.3) genes.

### Genotype–phenotype correlations in individuals with *RYR1* variants

The severity of clinical presentations in *RYR1*-related myopathy is influenced by the specific genetic variant (variant site and homozygous vs heterozygous state) [22, 23]. Given the strong association between genotype and phenotype [21, 24, 25], patients with heterozygous *RYR1* variants typically exhibit a less severe clinical picture compared to those with homozygous variants [26]. This correlation was illustrated in an analysis of a German family affected by MHS [16]. In this case, a patient (son) experienced an MH crisis during anesthesia, leading to the identification of a homozygous variant in *RYR1* associated with MH [16]. The patient’s sister also carried a homozygous variant, while the parents (mother and father) each possessed heterozygous variants. Consequently, MHS was diagnosed for the parents using the *in vitro* caffeine-halothane contracture test, the standard diagnostic procedure for MH and MHS [16]. Notably, the son and sister with homozygous variants exhibited greater contracture responses to both halothane and caffeine than their heterozygous parents, suggesting that genotype (homozygous vs heterozygous) is a significant determinant of the MH phenotype. However, interpreting genetic tests can be complex, and most patients with MH carry a heterozygous *RYR1* variant [16, 23]. Homozygosity has only been reported for two patients with the Cys35Arg substitution and one patient with the Arg614Cys substitution [16, 24, 27]. Therefore, individuals with MH-associated *RYR1* variants, even in the heterozygous state, should be regarded as at risk for MH.

### Anesthetic agents implicated in triggering MH

Halogenated anesthetics are the primary direct triggers of the biochemical disturbances linked to MH in patients with *RYR1* variants, with succinylcholine serving as an independent indirect factor [28]. Notably, succinylcholine alone has been reported to trigger MH in approximately 15.5% of susceptible individuals [28]. This phenomenon may be attributed to its indirect effect on *RYR1*, as succinylcholine induces sustained muscle action potentials, while volatile anesthetic agents provide continuous stimulation throughout the anesthesia period.

### Masseter muscle rigidity as a sign of MHS

Masseter muscle spasm following succinylcholine administration is a concerning clinical sign that may indicate MHS or an underlying myopathy [4]. Patients exhibiting marked rigidity of the jaw muscles should be monitored overnight for at least 12 h [12]. Given the low probability of MH development after isolated use of succinylcholine, continuing anesthesia with intravenous agents is often preferred over the more conservative approach of abandoning the surgical procedure [4].

## Conclusion

We present a case of a patient harboring two genetic variants contributing to succinylcholine-induced masseter spasm and severe rhabdomyolysis. One variant is associated with *RYR1*-related myopathy, while the other prolongs recovery after succinylcholine due to *BCHE* deficiency. Although these variants have distinct clinical effects, their coexistence may lead to a synergistic interaction. Specifically, the susceptibility related to *RYR1* was likely exacerbated by prolonged skeletal muscle membrane depolarization resulting from delayed succinylcholine hydrolysis.

## Supplemental data

We provide simplified animations that illustrate the sequence of cellular mechanisms involved in physiologic muscle contraction and pathogenic muscle contraction observed in a patient with pathogenic variants in *RYR1* and *BCHE*.

**Animation File 1.**
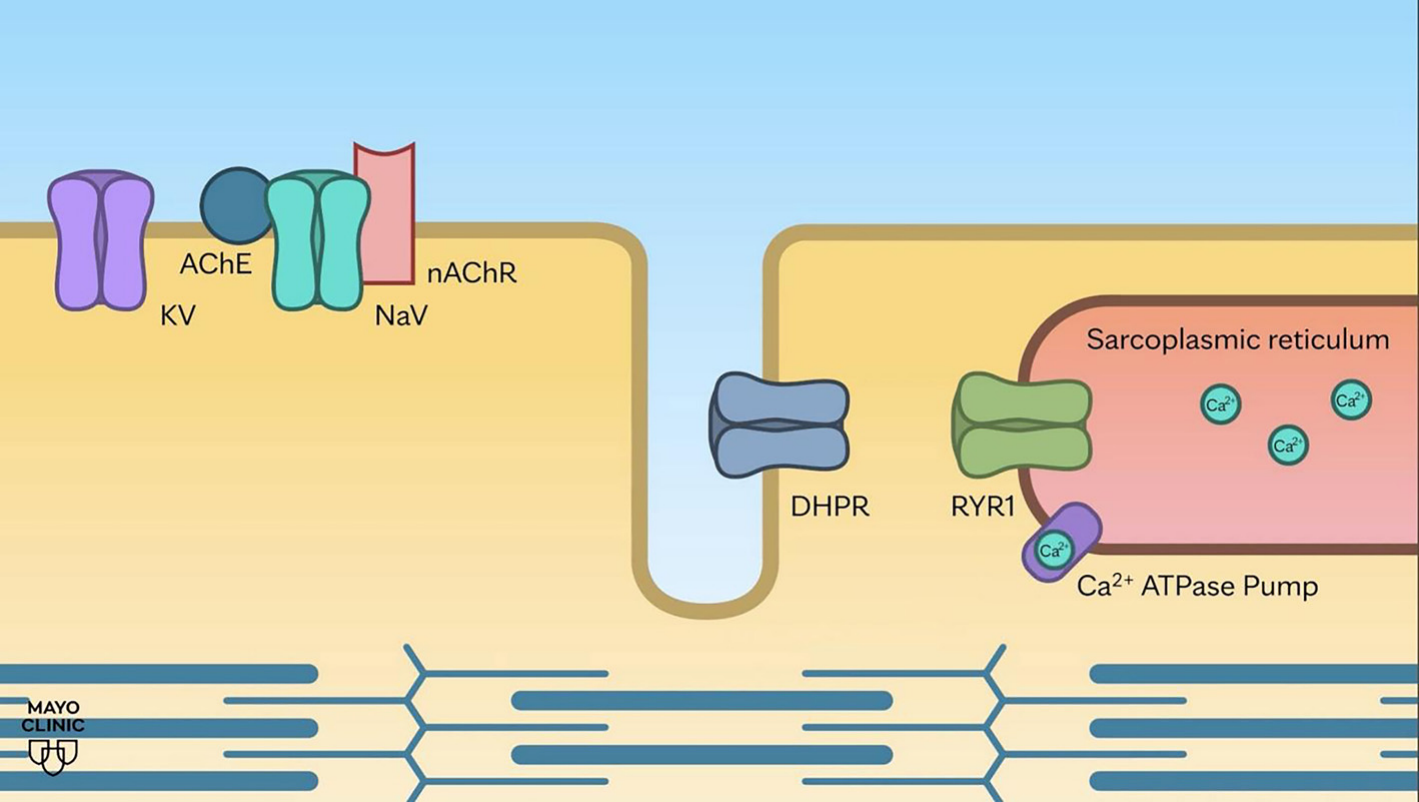
Scenario 1: Development of a normal action potential and the sequence of cellular events associated with muscle contraction and relaxation (YouTube: https://www.youtube.com/watch?v=55HEWeyNBck). (Used with permission of Mayo Foundation for Medical Education and Research.)

**Animation File 2.**
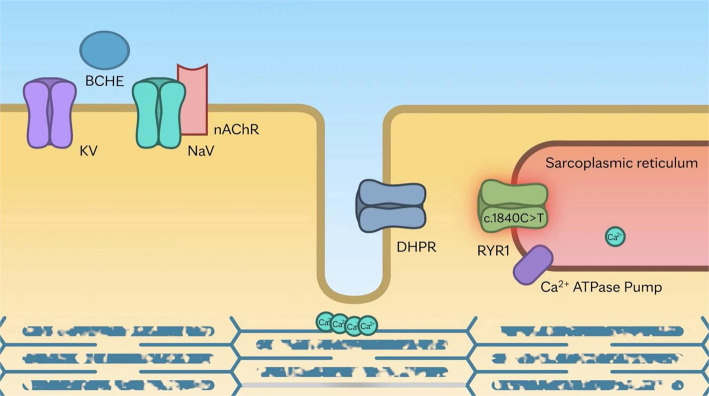
Scenario 2: Use of succinylcholine in patient with a pathogenic *RYR1* variant triggers prolonged myofilament stimulation resulting in myofibrillar disruption (YouTube: https://www.youtube.com/watch?v=worhWZhCsY4). (Used with permission of Mayo Foundation for Medical Education and Research.)

**Animation File 3.**
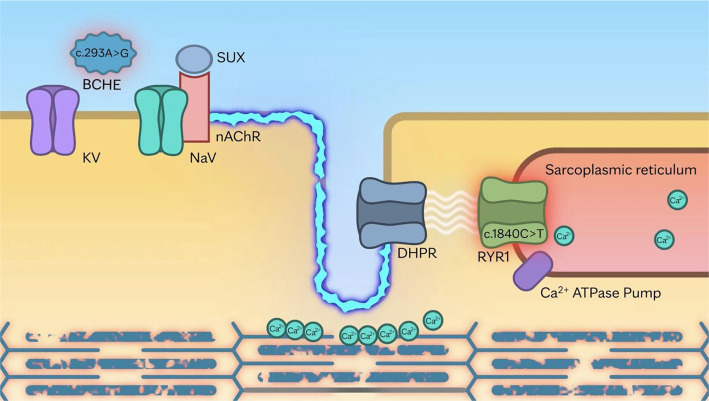
Scenario 3: Use of succinylcholine in a patient with butyrylcholinesterase deficiency and the pathogenic *RYR1* variant induces sustained depolarization, further extending myofilament stimulation and resulting in severe myofibrillar disruption with rhabdomyolysis (YouTube: https://www.youtube.com/watch?v=k-bY2cIRal0). (Used with permission of Mayo Foundation for Medical Education and Research.)
